# Total Femoral Replacement in Periprosthetic Femur Fracture: A Case Report

**DOI:** 10.7759/cureus.69087

**Published:** 2024-09-10

**Authors:** Alok C Agrawal, Lohitesh S, Harshal Sakale, Rudra Narayan Dash, Shivam Chauhan

**Affiliations:** 1 Orthopaedics, All India Institute of Medical Sciences, Raipur, Raipur, IND

**Keywords:** limb salvage, periprosthetic fracture, revision arthroplasty, total femur replacement, total hip replacement

## Abstract

Total femur replacement (TFR) is one of the most extensive endoprosthetic reconstruction procedures. The most common indication for the use of total femoral replacement is limb salvage in malignant bone tumors of the femur. This procedure is rarely performed outside the paradigm of oncological limb reconstruction. With the increased incidence of total hip and total knee replacements, complications of these procedures are also on the rise. Cases of complicated revision arthroplasties with severe bone loss, infection, and comminuted periprosthetic fractures may not have adequate residual bone stock for satisfactory fixation of megaprosthesis. With limited reconstruction options, most of these cases are offered lower limb amputation. TFR can be used as a limb salvage option in place of amputation in such cases.

There have been a few case reports of TFR for non-oncological indications in the literature. We present a case of periprosthetic comminuted distal femur fracture with a loose megaprosthesis following a road traffic accident (RTA), which was managed with TFR. At four years of follow-up, the patient showed good radiological as well as clinical outcomes.

## Introduction

With an increasing number of elderly patients undergoing arthroplasty surgeries and prostheses being used in more osteoporotic bone, periprosthetic fractures are becoming more common these days [1-3]. It can be challenging to treat periprosthetic fractures, as most patients require surgery to undergo internal fixation or revision of the prosthesis. Fixation of periprosthetic fractures is linked with a rather high risk of postoperative complications; 10-33% of patients need further surgery due to fixation failure, infection, or subsequent periprosthetic fracture. If further surgery becomes unsuccessful, they may eventually end up with amputation. That being said, endoprosthetic reconstruction (EPR) can be considered a better alternative procedure than lower limb amputation these days.

Although there are increasing indications for EPR in revision hip and knee arthroplasty, including severe bone loss, infection, and periprosthetic fractures, the conventional application of EPR has been restricted to extensive oncological resections around the hip and thigh. Therefore, it is expected that endoprosthetic revision surgeries will be progressively implemented in the foreseeable future instead of hip disarticulations enabling limb-salvage surgeries [4].

Total femoral replacement (TFR) is believed to be the most extensive surgical procedure. Dr. Buchman first reported this type of lower-extremity salvage arthroplasty procedure in 1965. By the 1980s, modular femoral prostheses had arrived, ushering in a modern era in orthopedic surgical oncology. Modular mega prostheses are made up of multiple components that may be put together in multiple ways to best repair a specific bone deficit. This also overcomes the manufacturing wait for the customized models, allowing surgery to proceed efficiently and on schedule. Furthermore, recent studies have demonstrated a reduced incidence of mechanical failure and need less effort for revision, as only the failed components have to be replaced during revision surgery [5].

Despite the apparent benefits of EPR in terms of mobilization with full weight bearing, a lot of uncertainty surrounds the long-term dependability of these complex surgeries, particularly with regard to infection, prosthesis failure, aseptic loosening, and periprosthetic femoral fractures (PFF). Furthermore, the primary source of failure in the subsequent years after surgery is PFF, which occurs in 10% to 24% of patients. Due to the level of difficulty of the procedure, a certain level of expertise and proficiency is required. The success of this technique also depends on the judicious selection of surgical patients and expectations. Several findings on functional evaluation following TFR in musculoskeletal tumors have been documented in the literature; however, only a few articles on non-oncologic patients exist [6].

It is worth mentioning that the management of periprosthetic femoral fractures around proximal femur endoprostheses could vary from standard joint replacement procedures, hence presenting an obstacle for orthopedic surgeons because of patient characteristics and implant variations [7,8]. These patients could also refuse to have revision surgery, which carries a significant risk of complications. To the best of our understanding, there is a dearth of information in the literature on the treatment of periprosthetic femur fractures around tumor endoprostheses.

In this study, we present a case of periprosthetic comminuted distal femur fracture with a loose prosthesis following a road traffic accident (RTA), which was managed with total femur replacement. Twenty-two years ago, this patient was operated on twice for a giant cell tumor of the proximal femur with curettage, cementation, plate osteosynthesis, and then long stem proximal femoral megaprosthesis. For this case study to be submitted for publication, the patient provided her informed consent.

## Case presentation

**Figure 1:**
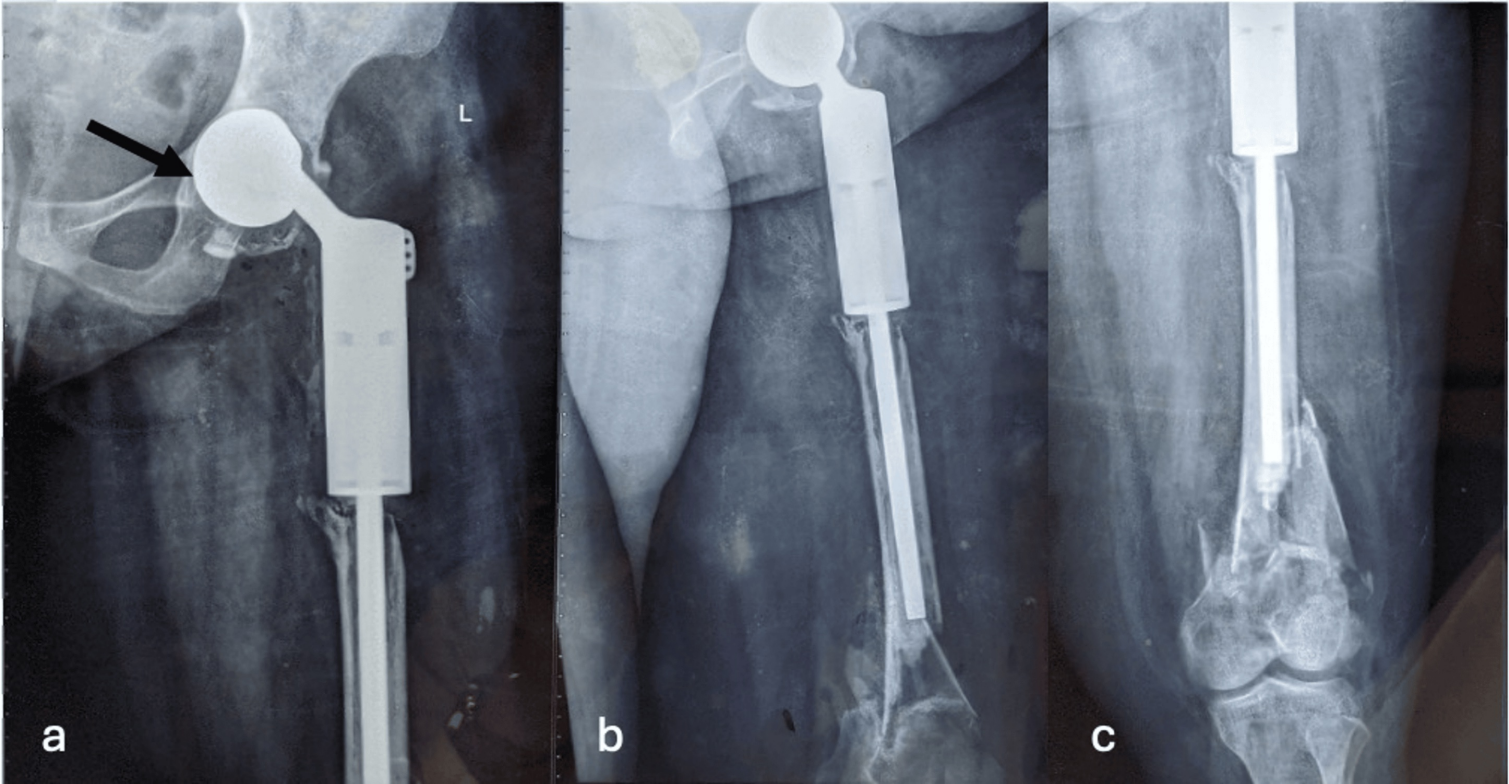
Preoperative X-ray of left side showing tumour megaprosthesis with comminuted periprosthetic distal femur fracture and acetabular component loosening a. X-ray(anteroposterior view) of the left hip and proximal femur, arrow shows acetabular loosening; b. X-ray(lateral view) showing the whole of the prosthesis and comminuted periprosthetic distal femur fracture; c. X-ray(anteroposterior view) of the distal femur showing comminuted periprosthetic distal femur fracture.

A 40-year-old female who was a homemaker presented with a bilateral open fracture of the distal femur following an RTA. In the past, the patient had a giant cell tumor of the proximal femur on the left side, which was initially managed with curettage, polymethylmethacrylate (PMMA) cement insertion, and plate osteosynthesis 21 years ago. Upon recurrence of the tumor, she underwent excision and total hip replacement with a long stem proximal femur megaprosthesis twenty years ago. The patient had also complained of left hip pain with weight bearing for two years. Following the trauma in RTA, a thorough clinical and radiological evaluation was performed, and a periprosthetic fracture of the distal femur on the left side with acetabular component loosening was confirmed (Figure 1).

Upon healing of the wound, the distal femur fracture on the right side was managed with osteosynthesis with a plate. On the left side, the patient underwent total femoral replacement using a constrained, uncemented acetabular component. At four years of follow-up, the patient is having a good functional outcome.

**Figure 2:**
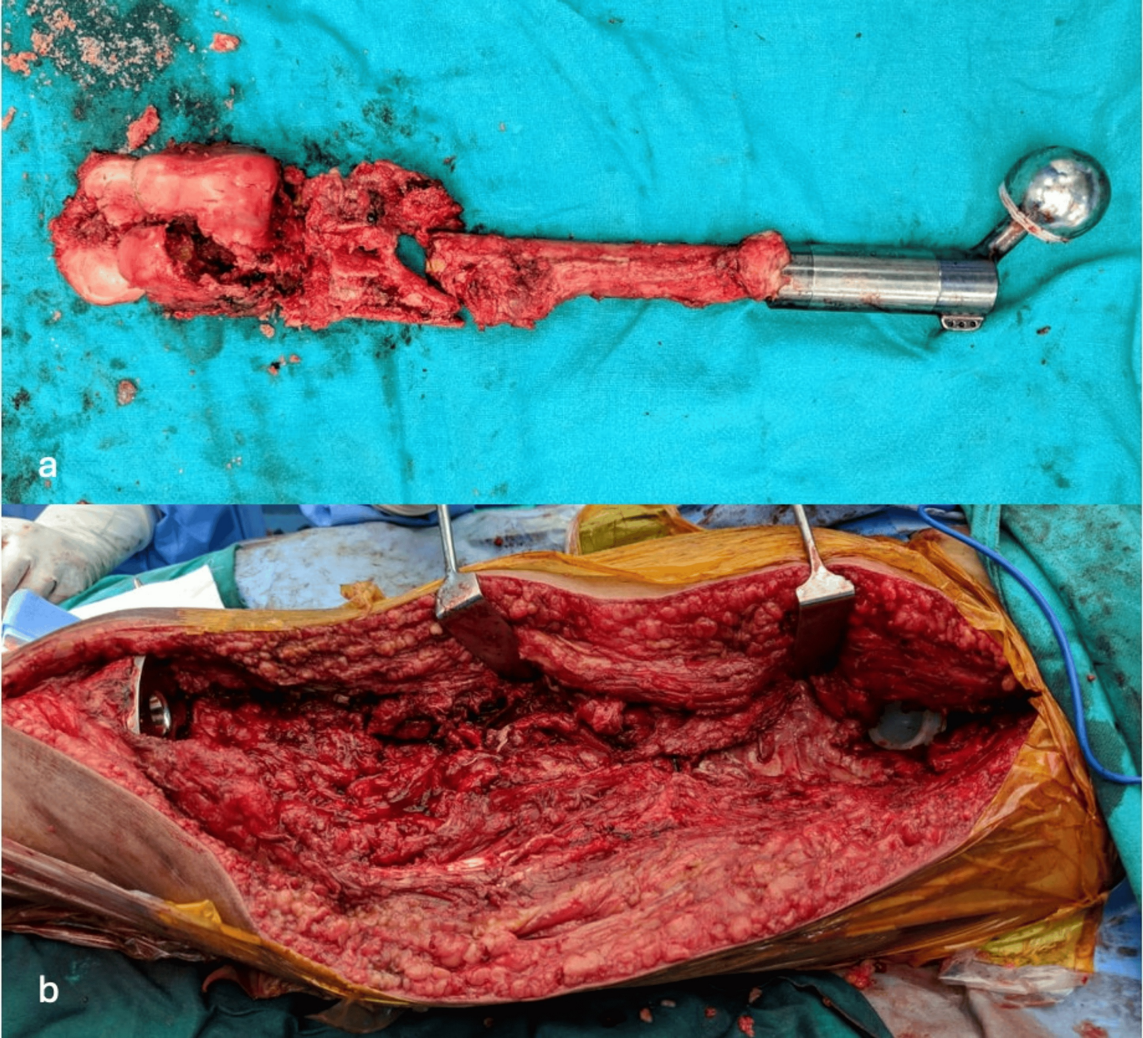
Removal of megaprosthesis and comminuted distal femur and preparation of acetabular and tibial site for total femur prosthesis placement a. Removal of megaprosthesis and comminuted distal femur; b. Preparation of acetabular and tibial site for total femur prosthesis placement.

The lateral decubitus posture was used to position the patient on the operating table. After cleaning the limb with a betadine-based surgical prep, the extremity was covered with a free drape. The incision was taken on the lateral aspect, some distance above the greater trochanter up to the tibial tuberosity. After splitting the gluteus maximus, the iliotibial (IT) band was cut along the direction of its fibers distally up to Gerdy’s tubercle. External rotators and capsules were cut to approach the hip joint. The prosthetic femoral head was dislocated, and the abductors that were attached to the femoral prosthesis were separated for later re-attachment to the prosthesis. The entire femoral prosthesis, along with the distal femur, is excised up to the knee joint (Figure 2).

**Figure 3:**
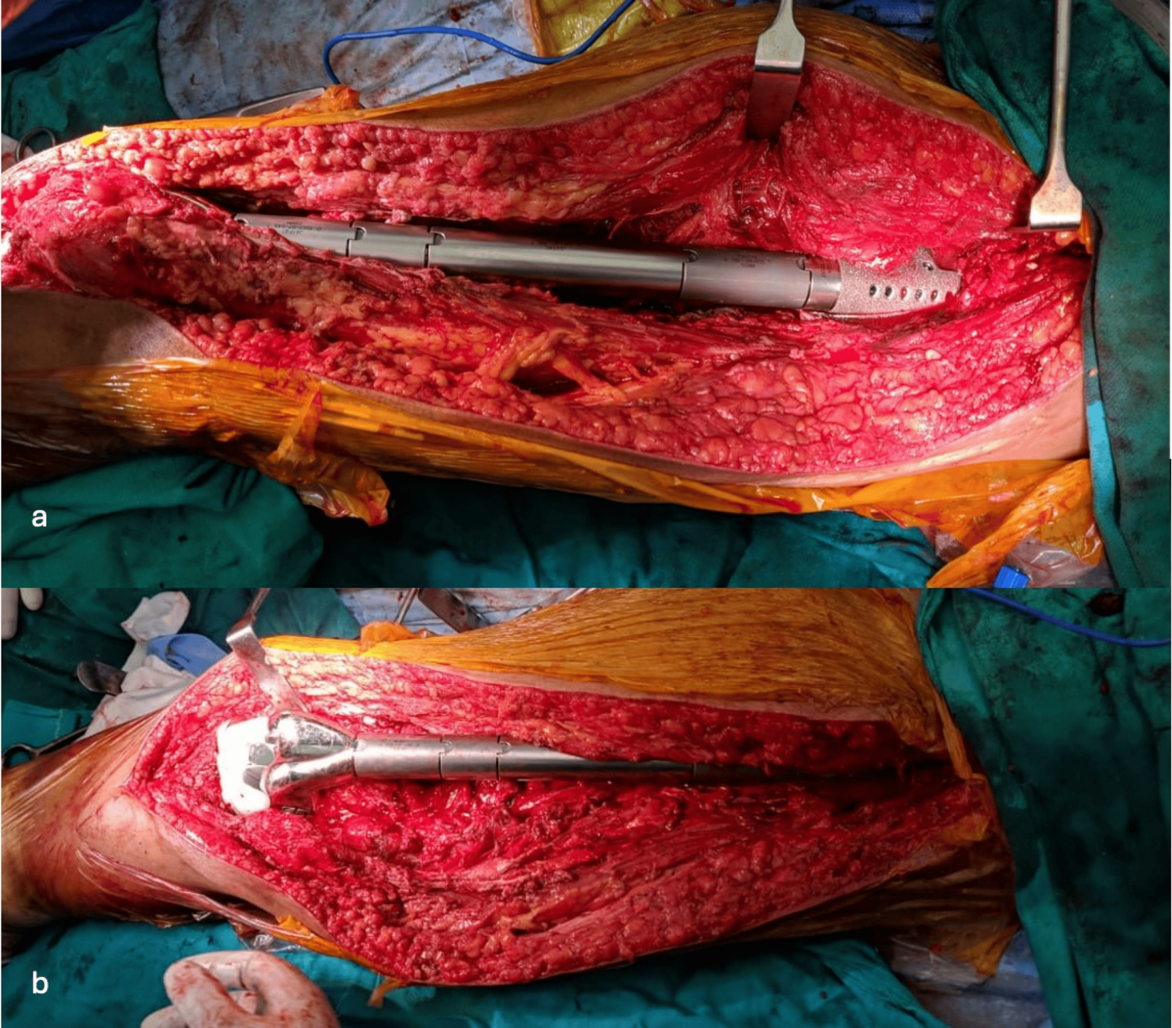
Total femur prosthesis placed, hip reduced and tibial hinged component connected a. Total femur prosthesis femoral head reduced into acetabular cup; b. The tibial hinged component connected.

After proper acetabular preparation in appropriate anteversion and inclination, a MODULOC (Adler Healthcare Pvt. Ltd., Maharashtra, India) bipolar cup is mounted. A total femoral replacement construct, RESTOR (Adler Healthcare Pvt. Ltd., Maharashtra, India) trial, was constructed based on previous X-ray measurements. The femoral length was confirmed with reference to the opposite limb on the table. After placing the prosthesis trail, the distal part was connected to the hinged knee component. The hip component was then reduced to the acetabulum. Range of motion and stability were verified. Once the trial appeared stable, the final prosthesis was placed, and the abductors were attached to the prosthesis (Figure 3).

Final stability was once again confirmed. The wound was then closed in layers. The rehabilitation procedures followed are generally similar to those reported in the literature, outlining the significance of quadriceps muscular strengthening in ensuring competency of the extensor mechanism.

**Figure 4:**
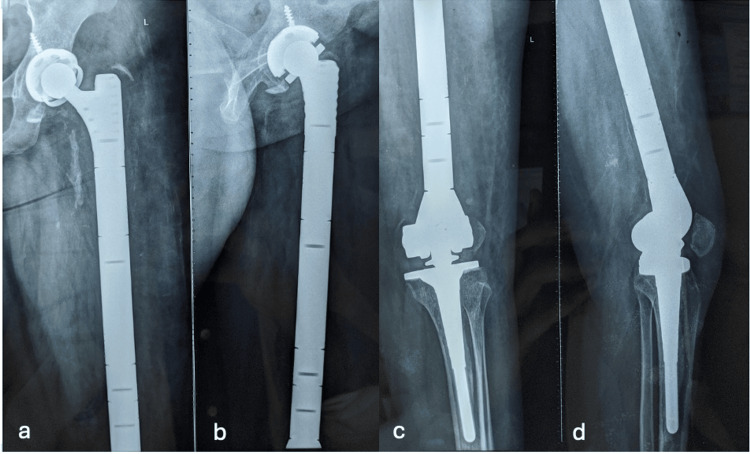
Four years follow up radiographs showing the constrained uncemented acetabular component and the total femur prosthesis a. X-ray (anteroposterior view) of hip showing constrained uncemented acetabular component; b. X-ray (lateral view) of hip showing constrained uncemented acetabular component; c. X-ray (anteroposterior view) of knee showing linked prosthesis component; d. X-ray (lateral view) of knee showing linked prosthesis component.

**Figure 5:**
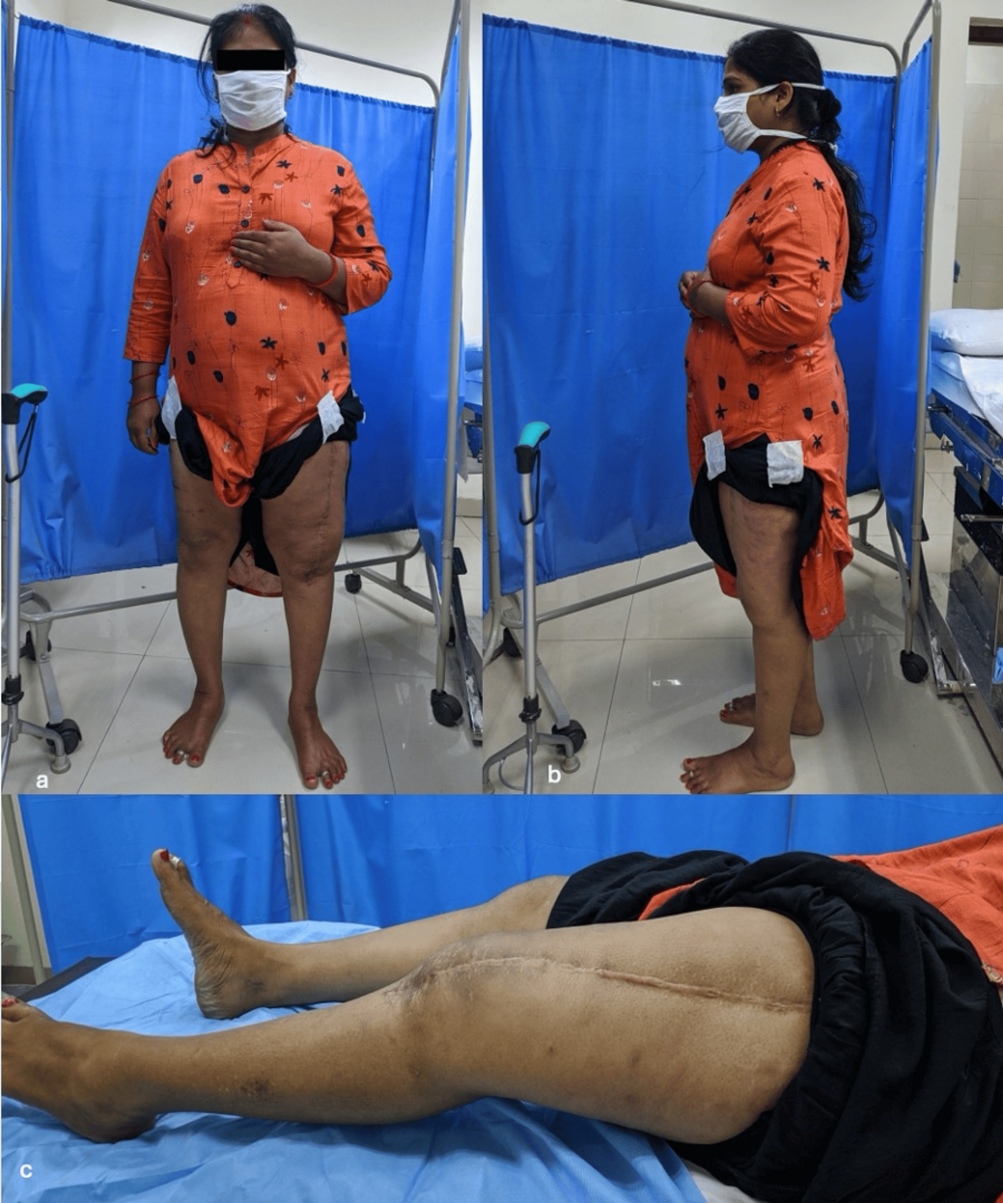
Clinical images at 4 years follow up a. Standing; b. Standing with healed scar on left thigh; c. Supine

Postoperative x-rays showed good prosthesis placement. The patient had good functional outcomes at six months and four years of follow-up (Figures 4, 5).

## Discussion

TFR is regarded as a limb salvage strategy for hip disarticulation and above-knee amputations in both oncologic and non-oncologic scenarios. When it comes to oncologic reconstruction for conditions including pleomorphic undifferentiated sarcoma, chondrosarcoma, osteosarcoma, and bony metastases that cause significant bone loss, TFR has a proven track record.

In addition to its well-known uses in tumor reconstruction, the TFR has been applied in non-tumor settings as revision arthroplasty rates continue to climb globally [9]. As the rationale for and number of hip and knee joint replacements performed each year increase, as does patient age, so does the prevalence of prosthetic disease in the form of septic and aseptic loosening, wear, osteolysis, and periprosthetic fracture [10,11,12]. Revision operations are becoming more common, contributing to a rise in complexity and a decrease in available bone stock for fixation. The end outcome may be a final-stage prosthetic disease with a severely damaged femur that is not tolerant of any known fixing or stabilization procedures and may eventually need total femur replacement.

Orthopedic oncologists have long utilized TFR as a means of limb salvage; therefore, the concept is not new. The first known TFR was carried out by Buchman in 1952 for the treatment of a large chondrosarcoma of the proximal femur [13]. Buchman is credited with performing the second TFR in 1965 to reconstruct a femur that was severely compromised due to Paget's disease [14]. Since then, there have been several cases of TFR registered for neoplastic conditions of the femur and limb-saving revision arthroplasty surgeries [15,16]. In another group of 32 total femur replacements in 28 patients, the biggest in English literature to date, Steinbrink et al. further clarified the indications for TFR [17]. Of the 32 cases, 21 did not involve tumors. Two nonunions, six periprosthetic fractures, three hips with extensive bone loss following aseptic loosening, and ten hips with extensive bone loss following several debridements due to infection all required total femoral arthroplasties. They reported on the follow-up status of 22 patients. Of the 11 patients in the series who had an infection, none had a recurring infection; however, two patients (18.2%) who were not infected developed an infection.

Our case describes a post-traumatic periprosthetic comminuted distal femur fracture with loose proximal femur megaprosthesis, which was managed with total femur replacement. When we opened the surgical site through a lateral approach, the prosthesis was found to be loose, and there was an extremely comminuted distal femur fracture, which was not feasible to reconstruct. No signs of infection or tumor recurrence were found during the intra-operative examination. So we opted for total femur replacement. The basic fundamentals of acetabular revision were followed during acetabular cup placement. Subperiosteal dissection is performed to minimize bleeding during femur excision. After placing the TFR prosthesis, proper reattachment of the abductors, which is the most essential component of the surgical procedure, is performed. Studies show 86 percent implant survival rates for comminuted periprosthetic fractures treated with TFR at a 10-year follow-up [18].

Immediate fixation, which enables early mobilization with early weight-bearing, is an intriguing advantage of TFR. TFR can enable people to walk again, albeit at a compromised level, and restore function. Still, this degree of function is preferable compared to what has been accomplished after hip disarticulation. In our case as well, we found that using TFR for periprosthetic fracture salvage resulted in a positive functional outcome in the form of significant alleviation of pain and improved overall knee society and hip score.

It is expected that TFR will take a considerably longer duration of operation than the majority of other revision arthroplasty surgeries due to the technical complexity of this surgery. This may raise the possibility of infection of the intra-operative wound and the ensuing periprosthetic joint infection, the most common complication. Owing to the level of complexity, a certain level of expertise and proficiency is required to perform the procedure. More volume of blood will probably be lost during the procedure as it involves extensive dissection. Nonetheless, using a subperiosteal dissection will lessen the bleeding.

## Conclusions

Periprosthetic femur fracture following proximal femur replacement remains a challenge for even the most experienced orthopedic surgeon. TFR is expected to become a more popular salvage option in the case of periprosthetic comminuted femoral fractures. For these patients to have the best long-term outcomes, careful patient selection, excellent surgical expertise, a thorough rehabilitation program, and prompt treatment of postoperative complications are required. A continued study with larger patient populations is required to better understand the functional results and problems associated with this procedure. In situations where reconstruction of comminuted periprosthetic fractures fails, amputation may be the only option.
